# Low carbohydrate high fat-diet in real life assessed by diet history interviews

**DOI:** 10.1186/s12937-023-00847-8

**Published:** 2023-03-02

**Authors:** Henrik Hagström, Linda Nyström Hagfors, Anna Tellström, Rikard Hedelin, Krister Lindmark

**Affiliations:** 1 Department of Public Health and Clinical Medicine, Umeå University, Umeå, Sweden; 2 Heart Centre, Umeå University Hospital, Umeå, Sweden; 3 Department of Food, Nutrition and Culinary Science, Umeå University, Umeå, Sweden; 4 Clinical Research Center, Umeå University Hospital, Umeå, Sweden; 5 Department of Clinical Sciences, Cardiology, Danderyd Hospital, Stockholm, Sweden

**Keywords:** Low carbohydrate diet, Low Carbohydrate High Fat, LCHF, Diet history interview, Saturated fatty acids, Cholesterol, Fiber

## Abstract

**Background:**

Low carbohydrate high fat (LCHF) diet has been a popular low carbohydrate diet in Sweden for 15 years. Many people choose LCHF to lose weight or control diabetes, but there are concerns about the effect on long-term cardiovascular risks. There is little data on how a LCHF diet is composed in real-life. The aim of this study was to evaluate the dietary intake in a population with self-reported adherence to a LCHF diet.

**Methods:**

A cross-sectional study of 100 volunteers that considered themselves eating LCHF was conducted. Diet history interviews (DHIs) and physical activity monitoring for validation of the DHIs were performed.

**Results:**

The validation shows acceptable agreement of measured energy expenditure and reported energy intake. Median carbohydrate intake was 8.7 E% and 63% reported carbohydrate intake at potentially ketogenic levels. Median protein intake was 16.9 E%. The main source of energy was dietary fats (72.0 E%). Intake of saturated fat was 32 E% and cholesterol was 700 mg per day, both of which exceeded the recommended upper limits according to nutritional guidelines. Intake of dietary fiber was very low in our population. The use of dietary supplements was high, and it was more common to exceed the recommended upper limits of micronutrients than to have an intake below the lower limits.

**Conclusions:**

Our study indicates that in a well-motivated population, a diet with very low carbohydrate intake can be sustained over time and without apparent risk of deficiencies. High intake of saturated fats and cholesterol as well as low intake of dietary fiber remains a concern.

## Background

Pamphlets on Low Carbohydrate Diets (LCDs) were published by William Banting already in the 1800s, but the use of these diets was popularized in the late 1960s and 70 s. The number of scientific publications regarding LCDs have been rising in recent years [1]. In Sweden, the Low Carbohydrate High Fat (LCHF) diet gained popularity around 2006. The LCHF diet has shown short term positive effects in reducing weight and in glucose control for diabetic patients [2, 3]. Sustainable effects on weight and glucose control beyond 12 months, as well as effects on cardiovascular risk are however uncertain [4, 5]. In addition to reducing the intake of carbohydrates, the LCHF diet encourages the intake of foods that are considered natural. Full-fat products are preferred over processed fat-reduced products. This often leads to a higher intake of saturated fatty acids than current evidence suggests to lower the risk of cardiovascular disease [6].

No universal definition of what constitutes a LCHF diet exists. To our knowledge, no exploratory study of diet habits among people who consider their diet to be LCHF has been published. A diet that excludes certain food groups may increase the risk of acquired deficiencies. LCHF diet limits the number of foods which could result in a lower intake of vitamins and minerals as well as a low intake of dietary fiber.

The loose definition and the wide variety of possible ways to compose a LCHF diet makes it a diverse diet with a range of possible benefits and risks. An exploratory study of nutritional composition in a population of self-claimed LCHF-eaters is therefore of interest.

The aim of this study was to evaluate the dietary intake in a population with self-reported adherence to a LCHF diet.

## Subjects and methods

### Study design

Cross sectional, observational study.

### Setting and participants

Volunteers were recruited through ads in a local newspaper in Umeå, Västerbotten county in 2017 and 2018. Applicants eligible for inclusion were at least 18 years old and they considered their diet to have been LCHF for at least 3 months. Exclusion criteria were current lipid-lowering medication, known familial hypercholesterolemia or inability to travel to study site. Participants were included consecutively as their application of interest reached the study e-mail inbox until one hundred participants were included. One hundred and fifty-two applicants were excluded. Reasons for exclusion were inability to travel to study site (*n* = 59), no response to invitation (*n* = 58), revoked interest in the study (*n *= 13), did not fulfill inclusion criteria (*n* = 20) and current lipid lowering medication (*n* = 2). Approval from the local Ethics Committee was obtained and all participants signed a written consent form.

### Variables and measurement

At the first visit, participants signed an informed consent form. Their weight, length, waist, and hip circumference were measured. Blood pressure and pulse were measured three times in a seated position using a manual aneroid sphygmomanometer. Smoking status, physical activity level, current medication and illnesses were reported by the participant in a questionnaire. Blood samples were taken for analysis of S-hemoglobin, S-sodium, S-potassium and S-creatinine as well as additional blood samples for further additional analyses. All measurements for all study subjects were taken by the same research nurse at the Clinical Research Center, Umeå University Hospital. The participants were fitted with a SenseWear Armband Pro3 (SWA) BodyMedia Inc., Pittsburgh, PA for 7 days, to assess total energy expenditure (TEE).

At the second visit, about 7 days later, diet history interviews (DHIs) were conducted with each participant. The DHI assessed food, alcohol and dietary supplement intake for a period of two weeks back in time from the interview date. The DHI has been validated previously [7]. The DHI was however modified slightly to suit the target group of this study. The interviews took place at the Clinical Research Center, Umeå University Hospital or the Department of Food and Nutrition, Umeå University and lasted on average two and a half hours. After establishing meal patterns on weekdays and weekends, each meal and between-meal snack were discussed in detail, with questions about food choices, frequencies, and portion sizes. The amounts and type of fat used in cooking was subject for extra attention. To estimate habitual portion sizes, we used “Portionsguiden”, a food photography atlas developed by the Swedish National Food Agency. Household measures and bags filled with seeds in different volumes as well as product information on the Internet, were also used to estimate portion sizes. To stimulate memory, a checklist of different foods was used. The participants were also asked if the weeks covered by the DHI were representative of their usual intake.

The reported food intake was converted into estimated energy and nutrient intake using the nutritional analysis software Nutrition Data (Nutrition Data Sweden AB) based on the Swedish National Food Administration’s food database as well as data from Finnish, Norwegian and American food databases relevant to the Swedish market. When participants ate their meal at a specific restaurant, the restaurant was contacted to obtain information on nutrient composition and portion size. Participants were also encouraged to submit detailed recipes for composite dishes. In total, 720 specific foods, recipes and dietary supplements were created for our study population and added to the food database. All interviews were conducted, compiled and nutritionally calculated by the same dietitian. The time for data collection and compilation was May 2017 to January 2019.

### Validation of the reported energy intake

The reported energy intake (rEI) was compared to total energy expenditure (TEE) assessed with SWA. The participants were asked if the period when the accelerometer was worn was representative of their usual level of physical activity.

Detection of implausible levels of rEI was made using the cut-off method described by Goldberg et. al [8]. Basal metabolic rate (BMR) was calculated using the Mifflin St Jeor method [9]. This method has shown a slightly better accuracy compared to other methods [10]. Food Intake Level (FIL) was calculated by dividing rEI by BMR. A lower and upper cut-off were then calculated for each individual based on the 95% confidence limits of the agreement between FIL and Physical Activity Level (PAL = TEE/BMR). The cut-offs calculated indicates whether the FIL is plausible, based on the PAL, number of dietary assessment days (14 days) and number of individuals (*n* = 1, since individual cut-offs were calculated). Participants with FIL outside of the lower and upper cut-off values were considered under- and over-reporters and the remaining participants were considered acceptable reporters. The Goldberg cut-off method assumes that the subjects are weight stable. The participants were therefore asked to estimate any weight change in the last 6 months and in the last month. Due to the cross-sectional design of this study, weight change was never measured, but this estimation made by the participants was taken into account in the validation of the rEIs. Adjustment of the Goldberg classification was made with respect to reported change in weight in the last month. Participants that reported a weight loss and were preliminary classified as under-reporters were re-classified as acceptable reporters and vice versa with over-reporters who gained weight.

### Statistical methods

Statistical analysis was conducted in IBM SPSS Statistics for Macintosh, Version 26.0. Armonk, NY: IBM Corp. Shapiro-Wilks’s test was used to assess normal distributions. Normally distributed data are presented as mean ± standard deviation (SD). Non-normally distributed data are presented as median (25–75 percentiles). Group comparisons were made with Mann–Whitney U test when independent sampled non-normally distributed data was analyzed, and Wilcoxon signed rank test when paired non-normally distributed data was analyzed. The significance level of type 1-error was set to < 5%.

Access to complete study data may be granted upon request.

## Results

### Study population

One hundred subjects were included. The vast majority were from Umeå and surrounding areas. A few participants traveled from other parts of Sweden. Demographics of the study population are shown in Table 1. Mean age was 49,5 years and ranged from 20- to 80 years, 62% of the participants were women. Mean BMI was 26,5 and none of the participants was a current smoker. A DHI was completed by all participants. For one of the participants the activity monitoring failed. None of the participants had any clinically relevant deviation of S-hemoglobin, S-sodium, S-potassium or S-creatinine.

**Table 1:** Demographics

Characteristics	*n* = 100
Age – years	49.5±13.7
Women—%	62
Body Mass Index – kg/m^2^	26.5±5.4
Waist Hip Ratio	0.89±0.08
Blood Pressure Systolic – mmHg	125.4±18.3
Blood Pressure Diastolic – mmHg	82.6±9.8
Smokers—%	0

Data presented as mean values ± SD or percentages

### Validation of the dietary assessment

Out of the 100 participants, 73 considered the period assessed by the DHI to have been representative of their usual dietary intake. Twenty-five participants said that the period deviated slightly from their usual eating habits and two did not consider the assessed period to be representative of their normal dietary pattern.

Forty-five participants reported unchanged weight in the last 6 months whereas 44 reported weight loss and 11 reported weight gain. Those who gained weight reported a median 3.0 (1.5–5.5) kg weight gain and those who reported weight loss lost a median of 4.5 (7.2–2.1) kg.

The period of the measurement of physical activity was considered representative by 63 participants, 34 said they are usually more active and 3 said they are usually less physically active. Levels of FIL below the Goldberg cut-off were detected in 25 of the participants. Intake above the upper cut-off level was found in 3 of the participants. Acceptable level of FIL was found in 71 of the participants. When adjustment for reported weight change was made, 13 participants were classified as under-reporters, 3 as over reporters and 83 as acceptable reporters.

In one participant the activity monitoring failed, and that participant was excluded from the analysis of Goldberg levels as well as PAL and FIL/PAL.

Reported energy intake and TEE are shown in Table 2. Median daily rEI for the acceptable reporters was 8.6 (2054) MJ (kcal), 7.8 (1864) MJ (kcal) for women and 10.2 (2437) MJ (kcal) for men. The rEI was significantly lower than the TEE for acceptable reporters as well as the whole study population (*p* < 0.001). Pearson correlation for rEI and TEE was found to be 0.446 for the whole group and 0.507 for acceptable reporters (*p* < 0.001).

**Table 2:** Reported energy intake and measured energy expenditure

Median (25–75 percentiles)	All (*n* = 100)	Acceptable reporters (*n* = 83)
Energy Intake, EI, kcal)	2007 (1570-2422)	2054 (1657-2491)
Total Energy Expenditure, TEE kcal	2410 (2097-2938)	2372 (2082-2853)
Food Intake Level, FIL)	1.3 (1.1-1.7)	1.4 (1.1-1.7)
Physical Activity Level, PAL (SWA)	1.5 (1.4-1.7)	1.5(1.4-1.6)
FIL/PAL	0.86 (0.69-1.04)	0.90 (0, 75-1, 05)

*N* = 99 for TEE, PAL and FIL/PAL due to one failed activity monitoring

**Fig. 1:**
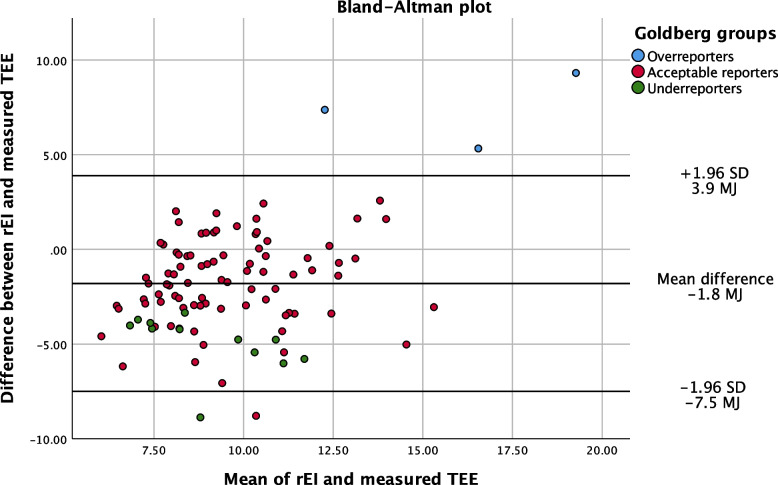
Bland–Altman plot. Legend: Underreporters *n* = 13, Acceptable reporters *n* = 83, Overreporters *n* = 3

Agreement between rEI and TEE was analyzed with a Bland–Altman plot shown in Fig. 1. The Bland–Altman analysis shows a mean difference of 1.8 MJ (427 kcal) and the distribution is fairly centered around the mean.

Median FIL was 1.31 (1.07–1.67) in the population. The participants that reported weight loss or weight gain in the past 6 months had median FIL of 1.15 (0.89–1.53) and 1.25 (0.88–1.55) respectively and among those that reported unchanged weight, median FIL was 1.57 (1.27–2.05). Both groups that reported change in weight had a significantly lower FIL compared to those with unchanged weight (*p* < 0.001).

Median PAL measured with SWA was 1.5 (1.4–1.7). Male participants had a significantly higher median PAL of 1.6 (1.5–1.8) compared to 1.5 (1.4–1.8) for women (*p* = 0.005). No difference was found between groups of weight change or groups of representative periods of measurement.

The intake levels presented will refer to the total study population unless otherwise stated. In Tables 3, 4, and 5, levels for both acceptable reporters and the study population in total are presented.

**Table 3:** Macronutrients

Median (25–75 percentiles)	All (*n* = 100)		Acceptable reporters (*n* = 83)		Recommendation	
	g/day	E%	g/day	E%	g/day	E%
Protein	82 (65.6–103)	16.9 (14.4–19.2)	83.3 (67.9–103.5)	17.0 (14.9–19.2)		10–20
Fat	156.1 (124.1–194.1)	72 (67.3–76.3)	161.5 (126.6–195.7)	72.3 (67.8–76.2)		25–40
Carbohydrates	42.9 (32.3–54.1)	8.7 (6.4–11.2)	44.6 (33.3–53.9)	8.7 (6.1–11)		45–60
Alcohol	4.3 (0–10.4)	1.4 (0.0–3.7)	3.7 (0–10.1)	1.3 (0.0–3.5)	< 10/20	< 5
Added sugar	1.8 (0.5–3.8)	0.3 (0.1–0.8)	1.8 (0.5–3.3)	0.3 (0.1–0.7)		< 10
Fiber	12.9 (8.3–17.5)		13.0 (8.3–17.5)		> 25–35	
SFA	70.7 (53.3–94.9)	31.9 (28.0–38.6)	72.7 (54.9–95.4)	32.0 (28.1–38)		< 10
MUFA	52.5 (39.6–65.6)	23.6 (21.9–25.8)	54.9 (41.5–66.5)	23.6 (21.8–26.6)		10–20
PUFA	18.7 (13.6–24.8)	8.1 (6.7–10)	19.1 (14–25)	8.4 (6.7–10.3)		5–10
Cholesterol	0.7(0.5–0.9)		0.7 (0.5–0.9)			

*SFA*  Saturated fatty acids, *MUFA* Monounsaturated fatty acids, *PUFA* Polyunsaturated fatty acids. Recommendations according to Nordic Nutrition Recommendations 2012 [11]

**Table 4:** Micronutrients, all participants

All (*n* = 100)	Without supplements				With supplements				Reference levels		
	Median (25–75 percentiles)	% of EAR	% below LI	% above UL	Median (25–75 percentiles)	% of EAR	% below LI	% above UL	EAR	Lower intake level (LI)	Upper intake level (UL)
		median				median			women/men	women/men	both sexes
Vitamin A – RE/day	1203.5(863.5–1644)	218	1	5	1297(988.3–1749)	252	0	5	500/600	400/500	3000
Vitamin D – μg/day	6.6(4–8.6)	88	7	1	12.6(6.6–52.6)	168	4	12	7.5	2.5	100
Vitamin E – mg/day	18.5(15.1–24.7)	355	0	0	20.9(16.2–29.2)	397	0	0	5/6	3/4	300
Vitamin K – μg/day	149.9(102.6–245.9)	n/a	n/a	n/a	158.6(109.6–251.7)	n/a	n/a	n/a	n/a	n/a	n/a
Vitamin B1 – mg/day	1(0.7–1.3)	96	4	n/a	1.1(0.8–2.5)	111	2	n/a	0.9/1.2	0.5/0.6	n/a
Vitamin B2 – mg/day	1.4(1.1–1.9)	116	5	n/a	1.7(1.2–3.1)	147	4		1.1/1.4	0.8	n/a
Vitamin C – mg/day	90(62.3–136.3)	169	0	2	126.5(80–220.5)	241	0	11	50/60	10	1000
Niacin – NE/day	36.1(30.1–42.3)	281	0	0	43.1(31.2–55.4)	308	0	0	12/15	9/12	900
Vitamin B6 – mg/day	1.4(1.1–1.6)	107	6	1	2.1(1.3–5.4)	160	3	8	1.1/1.6	0.8/1.0	25
Vitamin B12 – mg/day	5.4(4.2–8)	387	0	n/a	6.6(4.5–12.1)	472	0	n/a	1.4	1	n/a
Folate – μg/day	330(259.5–411)	165	0	0	388.5(284–578.5)	194	0	4	200	100	1000
Iodine – μg/day	218(173–290)	218	0	0	239.5(182–312)	240	0	0	100	70	600
Phosphorus – mg/day	1323.5(1091.8–1715)	294	0	1	1346(1101.3–1747)	299	0	1	450	300	3000
Iron – mg/day	9.1(7.3–11.7)	97	11	0	9.6(7.4–14.8)	108	11	7	10/7	5/7	25
Calcium – mg/day	832.5(637.5–1106.5)	167	8	1	848.5(665.5–1142)	170	7	1	500	500	2500
Potassium – mg/day	2850.5(2293.3–3449.5)	n/a	n/a	17	2872.5(2293.3–3474.8)	n/a	n/a	20	n/a	n/a	3700
Magnesium – mg/day	323(258.5–414.8)	n/a	n/a	n/a	439.5(313.75–612.5)	n/a	n/a	n/a	n/a	n/a	n/a
Sodium – mg/day	2783.5(2309.3–3705.5)	n/a	n/a	n/a	2819(2364.8–3737)	n/a	n/a	n/a	n/a	n/a	n/a
Selenium – μg/day	64.8(45.7–80.4)	192	0	1	76.1(48.9–108.7)	237	0	2	30/35	20	300
Zinc – mg/day	10.8(9.0–13.8)	200	0	2	13.6(9.6–19.7)	250	0	15	5/6	4/5	25

Recommendations according to Nordic Nutrition Recommendations 2012 [11]

**Table 5:** Micronutrients, acceptable reporters

Acceptable reporters (*n* = 83)	Without supplements				With supplements				Reference levels		
	Median (25–75 percentiles)	% of EAR	% below LI	% above UL	Median	% of EAR	% below LI	% above UL	EAR	Lower intake level (LI)	Upper intake level (UL)
		median				median			women/men	women/men	both sexes
Vitamin A – RE/day	1228(921–1655)	225	0	2	1301(1020–1713)	253	0	2	500/600	400/500	3000
Vitamin D – μg/day	6.6(4–8.7)	87	4	0	12(7.1–48.6)	159	2	12	7.5	2.5	100
Vitamin E – mg/day	18.6(15.4–24.9)	358	0	0	20.5(16.7–29.2)	400	0	0	5/6	3/4	300
Vitamin K – μg/day	153.3(106.5–247)	n/a	n/a	n/a	172.1(110.1–252.2)	n/a	n/a	n/a	n/a	n/a	n/a
Vitamin B1 – mg/day	1(0.8–1.3)	98	2	n/a	1.1(0.8–2.1)	112	1	n/a	0.9/1.2	0.5/0.6	n/a
Vitamin B2 – mg/day	1.5(1.2–1.9)	119	3	n/a	1.7(1.2–2.7)	149	3	n/a	1.1/1.4	0.8	n/a
Vitamin C – mg/day	89(62–134)	166	0	1	124(72–205)	232	0	6	50/60	10	1000
Niacin – NE/day	36.6(30.7–45.2)	282	0	0	43(31.3–53.7)	308	0	0	12/15	9/12	900
Vitamin B6 – mg/day	1.4(1.1–1.6)	108	4	0	2.1(1.4–5.1)	152	2	6	1.1/1.6	0.8/1.0	25
Vitamin B12 – mg/day	5.4(4.2–7.8)	388	0	n/a	6.6(4.7–11.9)	473	0	n/a	1.4	1	n/a
Folate – μg/day	332(261–402)	166	0	0	383(287–573)	192	0	3	200	100	1000
Iodine – μg/day	217(173–290)	217	0	0	249(187–309)	249	0	0	100	70	600
Phosphorus – mg/day	1370(1125–1720)	304	0	0	1379(1132–1764)	306	0	0	450	300	3000
Iron – mg/day	9.2(7.7–11.8)	69	8	0	9.7(7.9–14.4)	108	8	5	10/7	5/7	25
Calcium – mg/day	849(672–1127)	170	4	0	916(692–1166)	183	4	0	500	500	2500
Potassium – mg/day	2866(2332–3448)	n/a	n/a	14	2879(2332–3456)	n/a	n/a	17	n/a	n/a	3.7
Magnesium – mg/day	331(260–417)	n/a	n/a	n/a	442(330–611)	n/a	n/a	n/a	n/a	n/a	n/a
Sodium – mg/day	2783(2347–3645)	n/a	n/a	n/a	2816(2439–3698)	n/a	n/a	n/a	n/a	n/a	n/a
Selenium – μg/day	65.2(45.7–78.5)	193	0	1	76.2(51–105.3)	237	0	2	30/35	20	300
Zinc – mg/day	11(9.2–13.8)	203	0	1	13.8(9.8–19.6)	250	0	11	5/6	4/5	25

Recommendations according to Nordic Nutrition Recommendations 2012 [11]

### Composition of macro and micronutrients

**Fig. 2:**
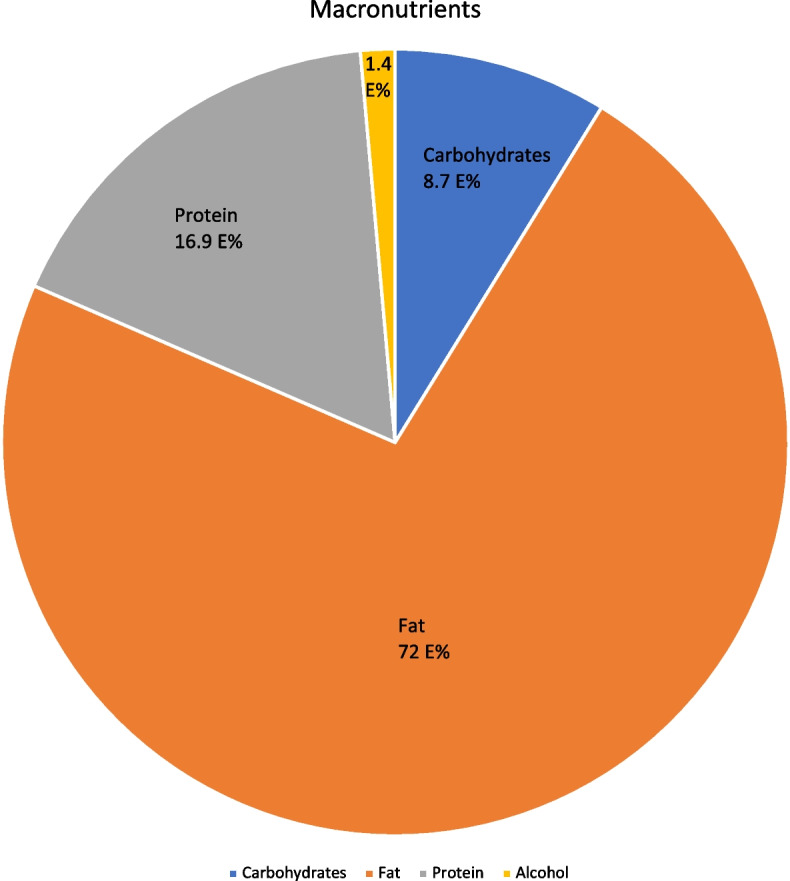
Macronutrients pie chart. Legend: Mean macronutrient energy intake as percentage of total energy intake

The median intake of fat was 72 E%. For protein, median intake was 16.9 E % and for carbohydrates 8.7 E% (Table 3 and Fig. 2). Saturated fatty acids comprised 31.9 % of the total energy intake. The most abundant n-3 and n-6 PUFAs were alfa-Linoleic acid and Linoleic acid, respectively. In the study group, median intake of n3-PUFA was 1.7 E%. Nine participants did not reach the recommended minimum of 1 E% n-3 PUFA. When considering only acceptable reporters, no change in median intake was found and five remained below 1 E% n-3 PUFA.

Sixty-six participants had a carbohydrate intake lower than 10 E%, 30 of these were however classified as non-acceptable reporters. Sixty-three participants had a reported intake of less than 50 g of carbohydrates per day and out of these, 49 were in the acceptable reporter group. Added sugar comprised in median 0.3% of participants’ total energy intake. Intake of dietary fiber was 12.9 g/day in median.

Micronutrient intake and reference levels are shown in Tables 4 and 5. In the whole population 76% used dietary supplements in some form, 74% for women and 79% for men. Estimated lower (LI) and upper intake levels (UL) as well as Estimated Average Requirement (EAR), available from Nordic Nutrition Recommendations 2012, were used to evaluate the risk for inadequate or exaggerated intake. Intake below the LI from diet alone was most common in iron, calcium and vitamin D. The instances of intake below LI, with or without supplements, were roughly cut by half when only acceptable reporters were analyzed. The instances of intake above UL were roughly cut by a third when only acceptable reporters were analyzed. When analyzing diet intake alone, median intake was below the EAR in three micronutrients: Vitamin-D, Vitamin-B1 and iron. The lowest intake compared to EAR was found in Vitamin D where median intake was 88% of EAR. Including supplements, none of the micronutrients had a median intake level below the EAR. Highest intake compared to EAR was found in Vitamin B12, both with and without supplements.

## Discussion

The dietary carbohydrate intake in our population was low and on levels that have been suggested to induce ketosis in most people [12, 13]. The substitution for carbohydrates was largely dietary fat. The levels of fat intake substantially exceed the recommended levels of the Nordic Nutritional Recommendations of 2012 [11]. The LCHF-diet encourages intake of food high in saturated fats which was also reflected in our population and may have potential long-term effects on cardiovascular health [14]. The median intake of cholesterol in our study population was 700 mg/day. This can be compared to the mean cholesterol intake of 293 mg/day reported in the United States [15] and 250–350 mg/day in the Nordic countries [13]. Cholesterol intake has been recommended to be below 300 mg/day in consensus statements over the years because of potentially adverse effects on the cardiovascular system [16–18]. In the current Nordic Nutritional Recommendations, no upper intake level has been set for cholesterol intake [13]. However, the recommendations regarding dietary patterns, including an increased intake of vegetables and a reduced intake of foods rich in saturated fat, should result in a reduced cholesterol intake.

A minimum intake of 1 E% of n3-PUFA is recommended because of its beneficial effects on risk for cardiovascular disease [11, 19, 20]. Median intake in our study was 1.7 E% and only a few of the participants had intakes below 1 E% n3-PUFA. This is probably an effect of the high intake of fat overall. Whether this ameliorates some of the negative effects of a high SFA-intake is however uncertain. It may be of interest that studies of native populations in Greenland and Canada with low incidence of cardiovascular disease revealed a high intake of SFA as well as n3-PUFA. These studies are however old and the settings very different from today [21].

The fiber intake was low which has been a target of criticism of the LCHF-diet. Greater intake of fiber has been associated with lower risk of cardiovascular disease, type-2 diabetes and cancer [22–24]. The relationship between dietary fiber intake and cardiovascular disease seems to be linear. According to Nordic Nutritional Recommendations, at least 25-35 g of fiber should be included in the diet each day [9]. In our study the intake was about half of that which may be associated with an excess risk. On the other hand, levels of added sugar in the diet were very low in our population, which is in line with nutritional recommendations and expected in a low carbohydrate diet population [11].

Our study population was largely normotensive which may be somewhat surprising. This might reflect a bias towards a healthy lifestyle in general in the volunteers in this study.

Supplements were frequently used in our population. Without supplements, vitamin D and iron were the micronutrients most commonly at intake levels at risk for deficiencies among acceptable reporters. The reported intake of vitamin D and Vitamin D-levels in the Swedish population has been estimated in the national dietary survey Riksmaten adults 2010–11 [25, 26]. The median intake levels of dietary vitamin D and intake from supplements were similar compared to our study. The frequency of intake levels below LI was also similar (9% in Riksmaten versus 7% in our study, not including supplements.) Considering only acceptable reporters, the level was 5%. In our study 38% of the participants met the AR of 7.5 μg/d compared to 33% in the Riksmaten sample. Nälsén et al. also analysed serum levels of vitamin D and found that despite that the majority did not meet the AR-level only 3% had serum levels below 30 nmol/L, which is often used to indicate risk of deficiency [24]. Significant seasonal variations were however present. Important sources of dietary vitamin D in the general population are often a big part of a LCHF-diet such as oily fish, dairy products and eggs. On the other hand, margarines enriched with vitamin D is probably less common. The present findings indicate that the intake of vitamin D among people who eat LCHF is in line with that of the general population.

Iron intake was also evaluated in Riksmaten adults 2010–2011 showing an intake of 10,4 mg/day in the general population, which is slightly higher than the intake reported in our study [26]. However, in our study 62% of the subjects were women. On average women have a lower intake of iron than men and in women of childbearing age the requirement is higher. Considering acceptable reporters in our population, 8% had an intake level below LI regardless of supplement intake. The clinical relevance of this is however unclear. No one in our population had anaemia or other obvious markers for micronutrient deficiency.

Intake levels above the recommended upper level were more common. Potassium, zinc and vitamin D had most participants above UL including supplements. Potassium intake at high levels is generally well tolerated if the subject is healthy. In the case of kidney disease or chronic medication, high intake may lead to hyperkalemia and serious adverse cardiac effects. High intake of zinc may induce copper deficiency as well as anemia, neutropenia and lower HDL concentrations. High Vitamin D intake may lead to hypercalcemia. In general, the intake levels of the micronutrients discussed are at high but not extreme levels and the risk of toxic effects are likely low [25]. In the blood sample analyses we found no indications of clinically relevant overconsumption of micronutrients. This is probably due to the absence of chronic disease in the population.

A strength with our study is that all diet history interviews were conducted with a validated interview form by the same dietitian and that rEI was validated using an objective measure of physical activity. We used a slightly modified version of the DHI to better suit our populations dietary pattern. This may affect the validity of the method. The validation of our results indicates a slight under-reporting of the rEI on a group level. In contrast, the few outliers in the Bland Altman plot suggest a possible overestimation of rEI in those with highest TEE. In most nutritional studies there is a discrepancy between caloric intake and measured energy expenditure [27]. Our data, however, reported a relatively low discrepancy and eighty-three of the one hundred participants were found to be acceptable reporters which strengthens the validity of the dietary assessment in this study.

The median FIL was higher among participants who reported unchanged weight, compared to those who reported weight loss or weight gain. FIL is expected to be lower among people who lose weight. On the contrary, people who gain weight should have a higher FIL than those who are weight stable, if the reported intake is accurate. However, underreporting has been shown to be associated with increased BMI as well as concerns about excess weight [28]. Regardless of the reason for misreporting, it is important to consider weight change when FIL is evaluated in relation to PAL, as this comparison is intended to be based on individuals who are in energy balance [29]. Therefore, we took weight change into account when accurate reporters where identified.

If the energy intake is under- or overreported, it is likely that the nutrient intake is under- and overestimated as well. This may lead to wrong conclusions about for instance inadequate nutrient intake. Therefore, the results in this paper are presented for acceptable reporters in addition to the results for all participants.

This study was observational and generalizing its findings should be made with caution. The study has several sources of bias. The recruitment was based solely on volunteers which probably selects the most motivated participants. We chose the participants own definition of LCHF because that mimics the real-world situation when a caregiver is confronted with the patient stating they follow a LCHF diet. The sample size is reasonably large and trying to capture a subpopulation like this in a larger screening study has a high risk of failure. Crude comparison of age, gender, BMI and WHR shows an acceptable agreement with the general population. Mean age was 49.5 with the youngest participant being 21 years old and the oldest being 80 years old. Mean age in Sweden was 41.2 years in 2018 [30]. Slightly more women (62%) participated. The study group was slightly overweight with a mean BMI of 26.5 kg/m^2^, which is comparable to the Swedish population [30, 31]. Overall, we found our study population to be fairly representative to the general population in Sweden with regards to baseline characteristics. With that said, any generalization of findings in our study should be made with caution. The study population was largely normotensive, and no participant reported smoking. This may be indicative for a bias towards a greater interest in health in our study population compared to the general population.

## Conclusions

Our study indicates that a LCHF diet can be sustained in real life. The reported carbohydrate intake level was generally very low and in well over half of the participants, the intake was at potentially ketogenic levels. The reduction in carbohydrates was mostly replaced with dietary fats. Dietary fiber intake was generally low. Although dietary supplements were common, most of the participants had adequate intake levels of micronutrients from their diet alone. Including supplements that was taken, it was more common that a participant had levels above the recommended upper intake level than below the lower intake level.

## Data Availability

The datasets used and/or analysed during the current study are available from the corresponding author on reasonable request.
